# Comprehensive Risk Assessment of High Temperature Disaster to Kiwifruit in Shaanxi Province, China

**DOI:** 10.3390/ijerph181910437

**Published:** 2021-10-04

**Authors:** Yining Ma, Suri Guga, Jie Xu, Jiquan Zhang, Zhijun Tong, Xingpeng Liu

**Affiliations:** 1 School of Environment, Northeast Normal University, Changchun 130024, China; mayn818@nenu.edu.cn (Y.M.); surgg146@nenu.edu.cn (S.G.); xuj463@nenu.edu.cn (J.X.); zhangjq022@nenu.edu.cn (J.Z.); gis@nenu.edu.cn (Z.T.); 2 State Environmental Protection Key Laboratory of Wetland Ecology and Vegetation Restoration, Northeast Normal University, Changchun 130024, China; 3 Key Laboratory for Vegetation Ecology, Ministry of Education, Changchun 130024, China

**Keywords:** risk assessment, high-temperature disaster, kiwifruit, climatic suitability zoning, hazard, vulnerability, exposure, disaster prevention and mitigation capacity

## Abstract

In recent years, the main kiwifruit producing region, central-south Shaanxi Province, has often suffered from the threat of extreme high temperatures. Assessing the risk of high-temperature disasters in the region is essential for the rational planning of agricultural production and the development of resilience measures. In this study, a database was established to assess the risk of a high-temperature disaster to kiwifruit. Then, four aspects, hazard, vulnerability, exposure and disaster prevention and mitigation capacity, were taken into account and 19 indexes were selected to make an assessment of the risk of a high-temperature disaster. At the same time, 16 indexes were selected for the assessment of the climatic suitability of kiwifruit in terms of light, heat, water, soil and topography, and were used as one of the indexes for exposure assessment. The analytic hierarchy process and the entropy weighting method were combined to solve the weights for each index. The results reveal that: (1) The Guanzhong Plain has a high climatic suitability for kiwifruit, accounting for 15.14% of the study area. (2) The central part of the study area and southern Shaanxi are at high risk, accounting for 22.7% of the study area. The major kiwifruit producing areas in Shaanxi Province (e.g., Baoji) are at a low risk level, which is conducive to the development of the kiwifruit industry. Our study is the first to provide a comprehensive assessment of the risk of a high-temperature disaster to the economic fruit kiwifruit, providing a reference for disaster resilience and mitigation.

## 1. Introduction

The first part of the Sixth Assessment Report (AR6) of the United Nations Intergovernmental Panel on Climate Change (IPCC) was released on 9 August 2021 [1]. The report states that the global surface temperature has increased by about 1.1 °C compared to 1850–1900, a level of warming not seen since 125,000 years ago. As a large agricultural country, China is experiencing extreme heat and weather caused by global warming [2], which has serious impacts on agro-ecosystems and national economic security, with losses increasing year by year [3,4,5]. The fruit tree industry, as an important part of agriculture, is often more vulnerable to extreme hot weather [6,7]. Therefore, reducing the impact of a high-temperature disaster on fruit tree production is of great importance in developing the agricultural economy, ensuring the supply of fruit and generating income and foreign exchange.

Shaanxi Province is the main production area of kiwifruit in China, with the highest planting area and yield year-round [8,9]. In 2019, the planting area of kiwifruit in Shaanxi Province was 87.67 km^2^, the production input was 1980.9 yuan/mu, the economic income was 0.49 yuan/km^2^, and the output was 1,072,400 tons, accounting for 6.05% of the province’s fruit yield [10]. Frequent extreme high-temperature events have had a negative impact on the kiwifruit industry in Shaanxi Province [11]. June to August is the prime period for kiwifruit fruit growth. If suffering from high temperature at this time, it will accelerate transpiration of plants and evaporation of orchard soil water. When the fruit is suffering from the effects of high temperatures, the skin is sunken and easily becomes soft and rotten. Sunburned fruit are very susceptible to falling off and in severe cases this will lead to a significant reduction in yield. To reduce the adverse effects of high-temperature disasters on kiwifruit, we should actively think about the following issues: How do we reduce disaster risk? How can measures be taken to avoid disasters before they happen? How can we respond positively to disasters and minimize losses? With the continuous development of disaster science, risk assessment and risk management have become important research directions. An objective and reasonable risk assessment can help policy makers to prevent disasters and reduce disaster losses. It also plays an important role in agro-meteorological disaster insurance and crop production planning.

The risk assessment of agro-meteorological disasters in China started relatively late, and research on the risk assessment of economic fruit is lacking. On the one hand, compared with other major agro-meteorological disasters, such as drought [12,13], chilling injury [14,15] and waterlogging [16,17], high-temperature disasters have been relatively little studied. On the other hand, the current research is mainly concerned with field crops such as maize [18,19], wheat [20,21], rice [22,23], etc. More importantly, research methods are mostly based on vulnerability [24,25,26], the lack of risk assessments that integrate hazards, vulnerability, exposure and disaster prevention and mitigation capacity. Luo et al. consider four aspects in terms of hazards, vulnerability, exposure and disaster prevention and mitigation capacity. They proposed a grey cloud clustering model based on panel data to assess the agricultural drought disaster risk of Henan Province [27]. Liu et al. analyzed drought risk under different future scenarios and constructed a socio-economic risk model based on hazards, vulnerability and exposure. The study found that climate change will increase the risk of future droughts, with negative socio-economic impacts on countries [28]. In addition, an increase in the frequency and intensity of droughts can have a negative impact on global food security [29]. We can draw on the experience of previous studies to develop an indicator system and model for assessing the risk of high-temperature disasters in relation to fruit. The results of the study provide insight into the frequency, intensity and spatial and temporal patterns of high-temperature disasters. The aim is to effectively strengthen the preventive management of risks and to actively improve emergency mitigation measures.

As AR6 points out: “The future of the planet depends, in large part, on the choices that humanity makes today. Many of the most dire effects of climate change can still be avoided if aggressive action is taken now [1]”. This is perhaps what risk assessment is all about: rather than actively remedying a disaster after it has occurred, it is better to effectively prevent it before it arrives. Avoiding and reducing the occurrence of disaster as much as possible is the way forward for disaster risk management, not just limited to agriculture. After all, “The future is in our hands [1]”.

Our aims included the following: (1) We want to conduct an in-depth analysis of historical meteorological data in Shaanxi Province, based on the “Four Factors” theory. As far as possible, a more comprehensive range of factors was taken into account, leading to the selection of indexes for assessing the risk of high-temperature heat disasters. (2) We wanted to use climate suitability as one of the exposure indexes. A comprehensive consideration of light, heat, water, soil resources and topography has led to the construction of a more complete climate suitability zoning index system and the study of kiwifruit climate suitability zoning in the study area. (3) Building a risk assessment model for high-temperature disasters to kiwifruit. The weight of each index was determined using the combination weighting method. Conducting a high-temperature disaster risk assessment and mapping. The assessment results were also validated using historical disaster data. (4) The results of the study can provide a scientific basis for disaster prevention and mitigation of kiwifruit and for achieving stable yields and increased income.

## 2. Study Area and Data Sources

**Figure 1:**
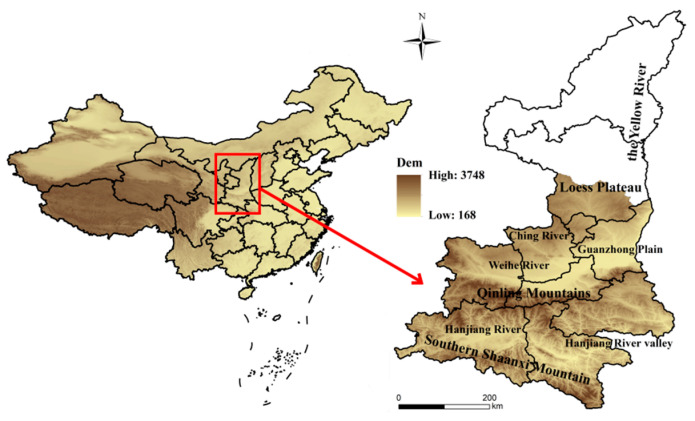
Location of the study area.

Shaanxi Province is located in the northwest of China (31°42′–39°35′ N, 105°29′–111°15′ E) (Figure 1). Within the territory of rolling hills and rivers, bounded by the Beishan Mountains and the Qinling Mountains, the province is divided into three major landform areas: the northern Shanbei Plateau, the Guanzhong Plain, and the Qinba Mountains. The average annual temperature is 13.0 °C, the average precipitation is about 576.9 mm and the frost-free period is about 218 days. Shaanxi Province straddles the northern temperate and subtropical zones and has an overall continental monsoon climate. The heat and water resources from south to north gradually reduce, and due to its unique climate and geographical conditions, kiwifruit is grown in most areas except northern Shaanxi.

Meteorological data that included daily observations of the maximum temperature, minimum temperature, average temperature, precipitation, relative humidity and gale days from 37 meteorological stations in Shaanxi Province were collected from the National Meteorological Information Center (http://data.cma.cn/ (accessed on 25 July 2021)) for the period from 1960 to 2020. Data that were abnormal or missing longer time series were removed to ensure the integrity of the data for that time period. Historical disaster data were obtained from the statistical yearbooks of Shaanxi and the China Meteorological Disaster Dictionary—Shaanxi Volume. Data on agricultural production conditions, socio-economics and kiwifruit planting situation by county in Shaanxi Province are from the 1990–2019 Statistical Yearbook of Shaanxi. Soil erosion data are from the Geographical Information Monitoring Cloud Platform (http://www.dsac.cn/ (accessed on 25 July 2021)). Soil data are from the China Soil Database (http://vdb3.soil.csdb.cn/ (accessed on 18 July 2021)).

## 3. Research Methods

### 3.1. Framework for High-Temperature Disaster Risk Assessment

**Figure 2:**
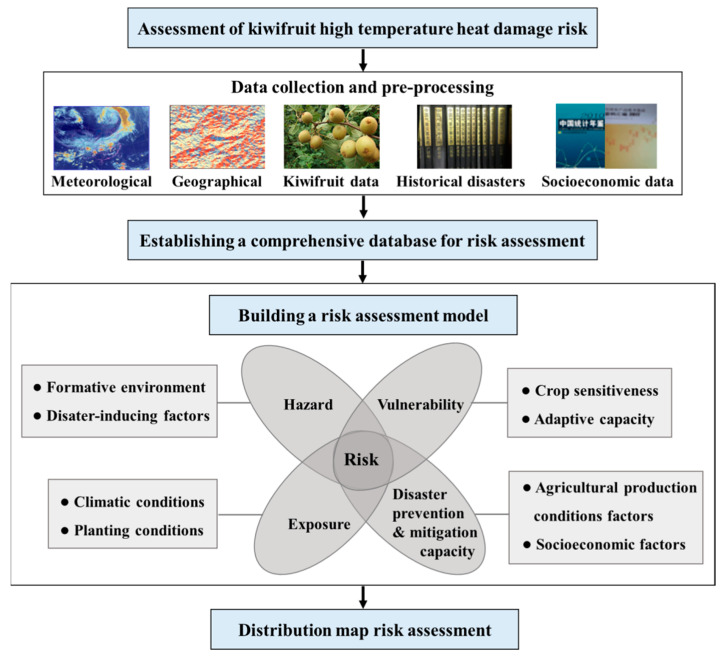
Kiwifruit high-temperature disaster risk assessment process for Shaanxi Province.

Figure 2 show the main research steps in the assessment of high-temperature disaster risk to kiwifruit in Shaanxi Province, including the following: (1) A comprehensive database for kiwifruit high-temperature disaster risk assessment was established by collecting relevant data such as meteorological data, historical disaster data and socio-economic data, etc. (2) Based on disaster risk assessment, the “Four Factors” theory, including selection of hazards, vulnerability, exposure and disaster prevention and mitigation capacity, was selected as the kiwifruit high-temperature disaster risk assessment method. The final 19 risk assessment indexes were selected, taking into account the environment of the study area and kiwifruit’s growth and development needs. (3) An analysis of the spatial and temporal distribution characteristics of hazards, vulnerability, exposure and disaster prevention and mitigation capacity was conducted. Then, relevant assessment indexes’ impact on the assessment of hazards, vulnerability, exposure and disaster prevention and mitigation capacity were analyzed. (4) Based on the above results, we conducted a comprehensive risk assessment and mapped high-temperature disaster to kiwifruit in Shaanxi Province and provide a scientific basis for decision making in response to high-temperature disaster.

### 3.2. Selection Risk Assessment Indexes

#### 3.2.1. Selection of Hazard Indexes

The selection of hazard indexes is based on both the disaster-inducing factors and the formative environment.

**Figure 3:**
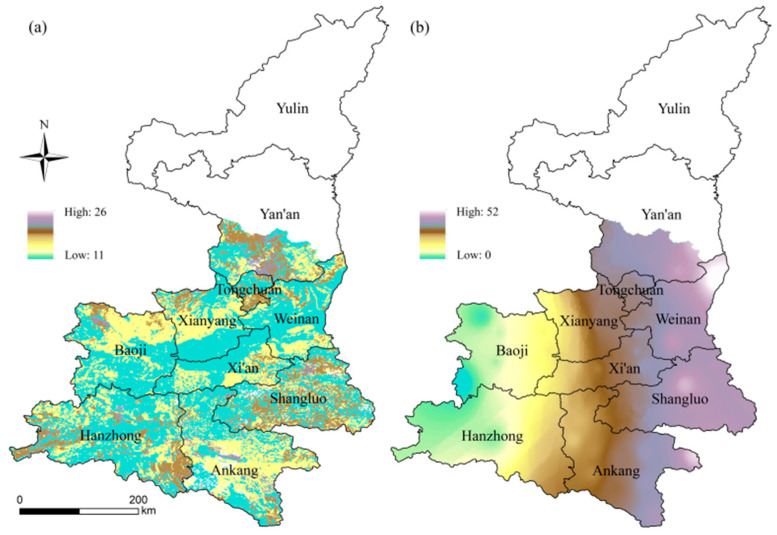
Spatial distribution of soil erosion (**a**) and gale days (day) (**b**).

(1) The disaster-inducing factors use the maximum daily temperature and duration from June to August to classify these into three levels, as shown in Table 1.

(2) Soil erosion (Figure 3a) and gale days (Figure 3b) were selected for the formative environment hazard index. Soil erosion, which reduces the amount of water available, increases the loss of nutrients from the soil and reduces the organic matter content of the soil, is one of the most serious threats to the world’s food production. Kiwifruit shoots are long and brittle, with large, thin leaves that are highly susceptible to high winds, causing branches to dry out and break. The severity of soil erosion and the duration of gale days are directly proportional to the hazard of the formative environment.

#### 3.2.2. Selection of Vulnerability Indexes

Vulnerability characterizes the degree of loss that may be caused by potential risks, based on both sensitivity and adaptability.

(1) Yield reduction rate (r) and yield reduction coefficient of variation (v)

Yield generally includes the trend yield, climate yield and random yield. The trend yield is determined by the level of social technology and the climate yield is influenced by climate factors. At the same time, variations in crop yields caused by changes in other factors are considered as random yield [30], calculated as follows:

(1) $$Y=Y_{t}+Y_{c}+Y_{e}$$

where Y is the actual unit yield (kg/hm^2^), $Y_{t}$ is the trend yield (kg/hm^2^), $Y_{c}$ is the climate yield (kg/hm^2^), and $Y_{e}$ is the random yield (kg/hm^2^), which is generally negligible. In this study, trend yields were calculated using the 3a sliding average method. Then, we introduced the concept of relative meteorological yield ($Y_{w}$). This is a comparable relative value that is not influenced by differences in the level of agricultural technology in different historical periods. It can reflect more effectively the fluctuations in the actual yield affected by meteorological disaster [31].

(2) $$Y_{w}=\frac{\left(Y-Y_{t}\right)}{Y_{t}}$$

A year with a negative relative meteorological yield is defined as a yield reduction year, and the meteorological yield reduction rate is calculated as follows:

(3) $$r=\frac{\sum^{}x_{i}}{n}$$

where $\sum^{}x_{i}$ is the sum of the negative relative meteorological yield and n is the total number of samples. r is used to describe the location of the concentration of negative values in the relative meteorological yield, i.e., the concentration of the years of yield reduction, which characterizes the average level of yield reduction subject to natural risk for that subject. The higher the rate of meteorological yield reduction, the higher the degree of damage caused by the disaster, and vice versa, as shown below:

(4) $$v=\sqrt{\frac{\sum^{}{\left(X_{i}-r\right)}^{2}}{\left(n-1\right)}}/r$$

where v is the meteorological yield reduction coefficient of variation and $X_{i}$ is the annual relative meteorological yield from year to year.

(2) Probability occurrence of yield reduction rate (p)

The probability occurrence of the yield reduction rate (p) is the cumulative probability that the relative meteorological yield will be less than a certain threshold value. An analysis of the variation of kiwifruit’s actual unit yield by county in Shaanxi Province over the years shows that meteorological disasters often affect kiwifruit when relative meteorological yields reach 5%, resulting in large losses. In contrast, relative meteorological yields of <−10% are rare. Relative meteorological yields <5%, with a yield reduction rate >5% chance as a vulnerability assessment index, can reflect, to some extent, the strengths and weaknesses of the climatic conditions and the degree of occurrence of meteorological hazards in kiwifruit growing areas. In this study, SPSS was used to test the normality of the relative meteorological yield series for each county, with the majority of counties conforming to a normal distribution and samples that did not conform to a normal distribution being normalized. Therefore, using the sample mean (u) and the sample mean square error (σ) to establish a distribution function, it was calculated as follows:

(5) $$F\left(x\right)=\int_{-\infty }^{x}\frac{1}{\sqrt{2\pi }\sigma }e^{\frac{1}{2\sigma }{\left(x-u\right)}^{2}}\mathrm{dx}$$

where x is the relative meteorological yield. When x is less than the critical value of −5%, p is calculated as follows:

(6) $$p\left(x<x_{0}\right)=\Phi \left(\frac{x_{0}-u}{\sigma }\right)$$

(3) Meteorological sensitiveness index

The meteorological sensitiveness index is calculated from the climatic yield and climatic productivity. The calculation formula is as follows:

(7) Km=Yw/Yv

where Km is the meteorological sensitiveness index, Yw is the actual productivity (kg·hm^2^) of the year, and Yv is the climatic productivity (kg·hm^2^); the Thornthwaite Memorial model [32,33] is used to calculate the climatic productivity of crops.

(8) $$\mathrm{Yv}=30000\left(1-e^{-0.000956\left(V-20\right)}\right)$$

(9) $$V=\frac{1.05R}{\sqrt{1+\left(\frac{1.05R}{L}\right)²}}$$

(10) $$L=300+25t+0.05t^{3}$$

where 30,000 is the empirical coefficient, e = 2.718, V is the annual average evaporation (mm), R is the annual precipitation (mm), L is the annual average maximum evaporation (mm), and t is the annual average air temperature.

(4) Selection of adaptive capacity indexes

**Figure 4:**
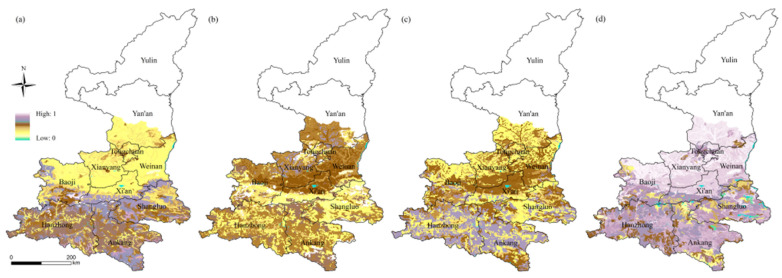
Spatial distribution of adaptive capacity indexes: (**a**) soil total nitrogen (g/kg), (**b**) soil total phosphorus, (**c**) soil total potassium content (g/kg) and (**d**) soil organic matter (g/kg).

Four indexes of adaptability were chosen (Figure 4): soil total nitrogen, phosphorus, potassium content and soil organic matter. The larger the index is, the stronger the soil adaptability is, and when the disaster occurs, it has stronger resistance and adaptability.

#### 3.2.3. Selection of Exposure Indexes

(1) Planted area as a proportion of the province’s planted area

(11) $$X_{A}=\frac{\mathrm{Ar}}{\mathrm{Aa}}$$

where $X_{A}$ is the proportion of kiwifruit planted area to kiwifruit planted area in Shaanxi Province. Ar is the area planted with kiwifruit in a county and Aa is the area planted with kiwifruit in the study area.

(2) Climate suitability

Agro-climatic zoning is a regional spatial classification that clarifies the relationship between climate and agricultural production according to the specific climatic requirements of agriculture and is an important basis for making full use of climatic resources and optimizing the structure and layout of agricultural cultivation [34,35]. Climatic suitability was chosen as one of the indicators of exposure; the higher the suitability, the higher the exposure and the greater the risk will be. Shaanxi Province is the number one kiwifruit producing region in China. The main problem facing the use of climatic resources in the region is the lack of indicators for the zoning of kiwifruit cultivation. To solve this problem, we use the ANUSPLIN software [36,37,38]. For the refined interpolation of each climate zoning indexes, 1 km × 1 km DEM raster data were used as covariates. Through the analysis of the demand for kiwifruit growth and development in the study area [39,40,41,42,43], we selected five major categories of light, heat, water, soil and topography, with 16 climate suitability assessment indexes (Table 2).

When zoning for climatic suitability, the not suitable areas are first eliminated according to the indexes to avoid compensatory effects between indexes. Subsequently, the climatic suitability regionalization indexes are scored with corresponding percentages according to the different zoning classifications, with the following formula:

For grid points located in a hardly suitable area:

(12) $$G=g_{\min}+\left(g_{\max}-g_{\min}\right)\times \frac{p-n_{\min}}{N_{\max}-n_{\min}}$$

For grid points located in a moderately suitable area:

(13) $$G=g_{\min}+\left(g_{\max}-g_{\min}\right)\times \frac{p-N_{\min}}{N_{\max}-N_{\min}}$$

For grid points located in a highly suitable area:

(14) $$G=g_{\min}+\left(g_{\max}-g_{\min}\right)\times \frac{p-N_{\min}}{n_{\max}-N_{\min}}$$

where G is the percentage scoring result of the grid points; $g_{\max}$ and $g_{\min}$ are the maximum and minimum values of the range of scores corresponding to that zoning level, respectively; the range of scores corresponding to the different zoning classifications is shown in Table 2; $N_{\max}$ and $N_{\min}$ are the maximum and minimum values of the corresponding criteria, respectively; $n_{\max}$ and $n_{\min}$ are the maximum and minimum values, respectively, in the data set of grid points corresponding to the zoning classification; p is the actual value of the grid point. After scoring, the results were summed according to a certain weighting to obtain an overall score for the climatic zoning of kiwifruit in the study area. The range of scores corresponding to the different zoning classifications is shown in Table 3.

The kiwifruit suitability assessment composite index ranges from 0 to 1. The higher the index, the higher the climatic suitability for kiwifruit planting. Climate suitability is classified into 4 classes according to the optimal partitioning method (Table 4).

#### 3.2.4. Selection of Disaster Prevention and Mitigation Capacity Indexes

Disaster prevention and mitigation capacity indicates the extent to which the study area can recover from a disaster in the long or short term. The higher the value, the less potential damage the study area may suffer and the lower the disaster risk.

### 3.3. High-Temperature Disaster Assessment Index System

The combination weighting method is used to determine the weight of each index, and the weighted comprehensive average method is used to construct risk assessment model (Table 5).

(15) $$H=\sum_{i=1}^{n}W_{\mathrm{Hi}}X_{\mathrm{Hi}}$$

where H denotes the hazard, which is the degree of natural variability that causes the disaster. The higher the value, the more severe the loss caused by the disaster and the higher the risk of the disaster. $X_{\mathrm{Hi}}$ and $W_{\mathrm{Hi}}$ represent the hazard index and the corresponding weight, respectively.

(16) $$S=\sum_{i=1}^{n}W_{\mathrm{Si}}X_{\mathrm{Si}}$$

(17) $$A=\sum_{i=1}^{n}W_{\mathrm{Ai}}X_{\mathrm{Ai}}$$

(18) V=S×(1−A)

where S and A denote crop sensitiveness and adaptive capacity, respectively, which are used to characterize vulnerability (V). $X_{\mathrm{Si}}$, $X_{\mathrm{Ai}}$, $W_{\mathrm{Si}}$ and $W_{\mathrm{Ai}}$ represent the assessment index and the corresponding

(19) $$E=\sum_{i=1}^{n}W_{\mathrm{Ei}}X_{\mathrm{Ei}}$$

where $X_{\mathrm{Ei}}$ and ${\;W}_{\mathrm{Ei}}$ represent the exposure index and the corresponding weight, respectively.

(20) $$C=\sum_{i=1}^{n}W_{\mathrm{Ci}}X_{\mathrm{Ci}}$$

where $X_{\mathrm{Ci}}$ and $W_{\mathrm{Ci}}$ represent the emergency response and recovery capability index and the corresponding weight, respectively.

### 3.4. High Temperature Disaster Risk Assessment Model

According to the “Four-Factors” theory of natural disaster risk formation, the four aspects of disaster are hazards, vulnerability, exposure and disaster prevention and mitigation capacity. We have established a kiwifruit high-temperature disaster risk assessment index to characterize the degree of hazard risk. The formula is as follows:

(21) $$R=H^{W_{H}}\times V^{W_{V}}\times E^{W_{E}}\times {\left(1-C\right)}^{W_{C}}$$

where R is the high-temperature disaster risk assessment index; H, E, V, C stand for for hazard, vulnerability, exposure and emergency response and recovery capability; W_H_, W_V_, W_E_, W_C_ are the weight for hazard, vulnerability, exposure and disaster prevention and mitigation capacity, respectively. The combination weighting method was calculated as 0.384, 0.203, 0.221 and 0.192.

### 3.5. Standardized Treatment of Assessment Indexes

Due to the different dimensions of each assessment index, the assessment index must be standardized in weight calculation to eliminate the influence of different units and different measures among the indexes. In this paper, the range method chosen [44] for positive impact indicators is:

(22) $$R=\frac{X_{i}-\min\left(X_{i}\right)}{\max\left(X_{i}\right)-\min_{j}\left(X_{i}\right)}$$

and for negative impact indicators it is:

(23) $$R=\frac{\max\left(X_{i}\right)-X_{i}}{\max\left(X_{i}\right)-\min\left(X_{i}\right)}$$

where R is the normalized index value, $X_{i}$ is the assessment indicator, $\max\left(X_{i}\right)$ is the maximum value in the sequence, and $\min\left(X_{i}\right)$ is the minimum value in the sequence.

### 3.6. Methodology

#### 3.6.1. Combination Weighting Method

(1) AHP method to determine subjective weights of indexes

The analytic hierarchy process (AHP) is a subjective weighting method that is suitable for quantitative analysis of qualitative problems under multi-criteria decision making and is commonly used in many fields [45].

(2) Entropy weight method to determine objective weights of indexes

The entropy method is an objective weighting method [46]. In information theory, entropy measures the amount of valid information provided by the data. If the information entropy of an indicator is lower, the more information the indicator has the more weight it will have in the evaluation [47,48,49].

(3) Combination weighting method

The combination weighting method combines the expert theoretical knowledge and rich experience (AHP method) with the full mining of data combination information of the objective weighting method (entropy weight method). To a certain extent, systematic and random errors can be reduced [50]. In order to scientifically assign weights to the combinations, reference is made to the principle of minimum discriminatory information. The objective function is defined as:

(24) $$\mathrm{minJ}\left(\omega \right)=\sum_{j=1}^{n}(\omega_{j}\ln\frac{\omega_{j}}{u_{j}}+\omega_{j}\ln\frac{\omega_{j}}{v_{j}})$$

$$s.t.\sum_{j=1}^{n}\omega_{j}=1,\;\omega_{j}\geq 0,\;j=1,2,\cdots ,n$$

Solving this optimization model yields the combined weights as:

(25) $$\omega_{j}=\frac{\sqrt{u_{j}v_{j}}}{\sum_{j=1}^{n}u_{j}v_{j}}$$

where $\omega_{j}$ is the combination weight and $u_{j}$ and $v_{j}$ are the subjective and objective weights of the indicator, respectively.

#### 3.6.2. Mann–Kendall Method

The M-K test was originally proposed and developed by H.B. Mann [51] and M.G. Kendall [52], and it is an effective tool recommended by the World Meteorological Organization for extracting trends in series variability. The principle of the method and the calculation steps are detailed in [53].

## 4. Results and Discussion

### 4.1. Comprehensive Assessment of High-Temperature Disaster Hazards

#### 4.1.1. Analysis of the Variation Characteristics of High-Temperature Events Frequency

**Figure 5:**
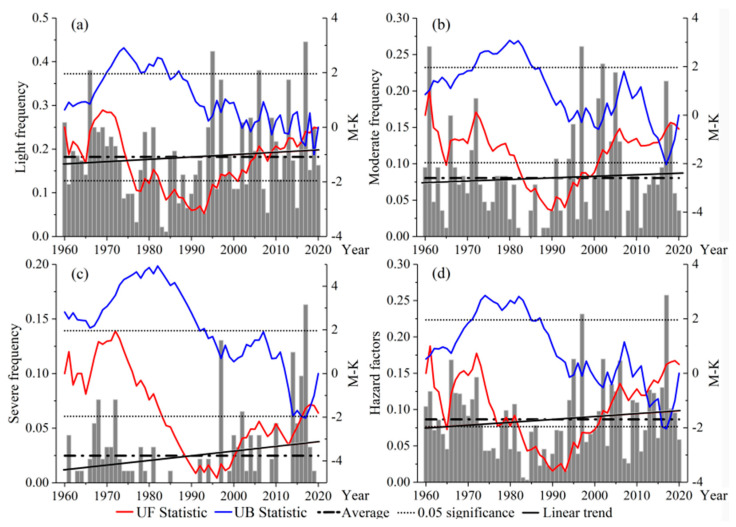
Variation and M-K test of the high-temperature events frequency and hazard index. (**a**) Light frequency. (**b**) Moderate frequency. (**c**) Severe frequency. (**d**) Hazard factors.

Figure 5 shows the frequency of light (a), moderate (b) and severe (c) high-temperature events in the study area from 1980–2020, respectively. It can be seen that the change in frequency fluctuates, with averages of 0.18, 0.07 and 0.02, respectively. During 1980–1990, there were few high-temperature events, and no severe high-temperature events occurred for many years. Figure 5d shows the trend and M-K test of the disaster-inducing factors hazard index. The change shows a “U-shaped” increasing and decreasing trend, with the minimum value occurring in 1984 (0.0032) and the maximum value in 2017 (0.26). The UF and UB curves cross in 1961 and 2012, indicating a sudden change in the hazard index during those two years. Overall, the UF and UB statistics are basically >0, indicating an upward trend in variation, which also indicates that the adverse effects of high temperature on kiwifruit are also increasing year by year.

**Figure 6:**
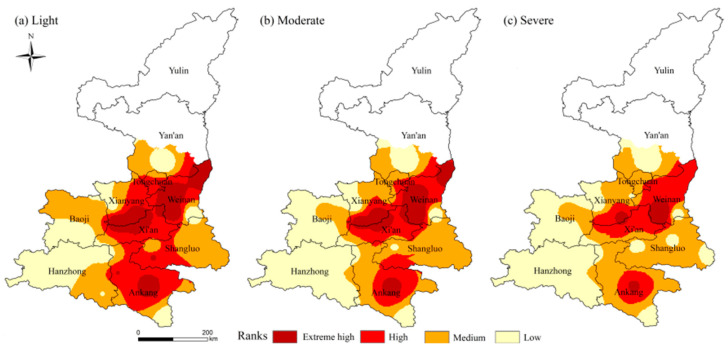
Spatial distribution of the frequency of high-temperature events.

Figure 6 shows the spatial distribution of the frequency of light (a), moderate (b) and severe (c) high-temperature events in the study area. The high incidence of severe high-temperature events was relatively low and relatively concentrated in central Ankang, southwestern Weinan and southern Xianyang. Moderate high-temperature events are concentrated in central Ankang, northern and south-central Weinan, and southern Xianyang where it meets the northwestern part of the Xi’an. Light high-temperature events of varying degrees of intensity occurred in all areas except central Hanzhong and southern Baoji. Spatially, severe high-temperature events have a smaller impact area.

#### 4.1.2. Analysis of Spatial Patterns of High-Temperature Disaster Hazard

**Figure 7:**
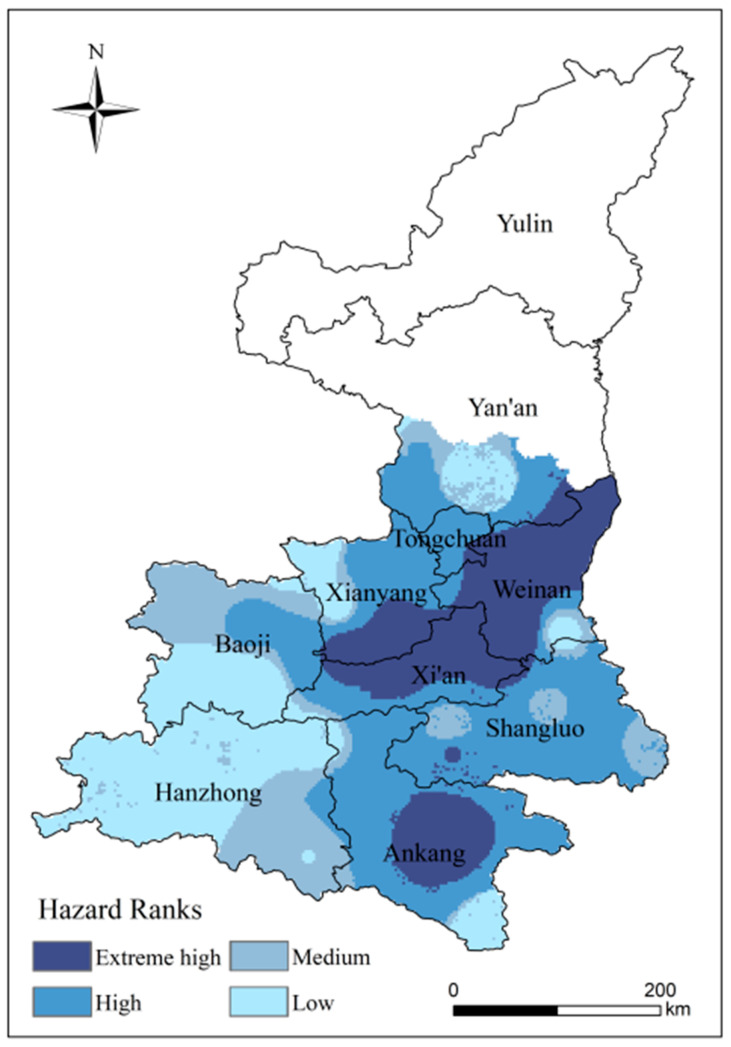
Distribution of hazard to kiwifruit in Shaanxi.

Figure 7 shows the spatial distribution of the hazard of high-temperature disaster in the study area. The spatial distribution is based on the Inverse Distance Weighted (IDW). The extreme high-hazard areas are concentrated in the southeast of the study area, influenced by the subtropical or warm temperate monsoon climate. Extreme high-hazard areas are concentrated in central Ankang, southern Xianyang, Xi’an and most of Weinan, accounting for 21.16% of the study area. High-hazard areas make up 37.72% of the study area, the largest area of any class. Covering the south-central Loess Plateau and the hinterland of the Guanzhong Plain. Most of these areas are major grain and fruit producing areas, and the extreme high-hazard areas are exposed to a higher frequency or intensity of high-temperature disasters, facing extreme high risk, and there are great potential economic losses. Areas of medium hazard are concentrated in the north of Baoji and parts of the border between Hanzhong and Ankang, accounting for 16.82% of the study area. The low-hazard areas are mainly in the south of Baoji and most of Hanzhong, and sporadically in Ankang, Weinan and Yan’an, accounting for 24.3% of the study area.

### 4.2. Comprehensive Assessment of High-Temperature Disaster Vulnerability

#### 4.2.1. Changes in Kiwifruit Yield by County

**Figure 8:**
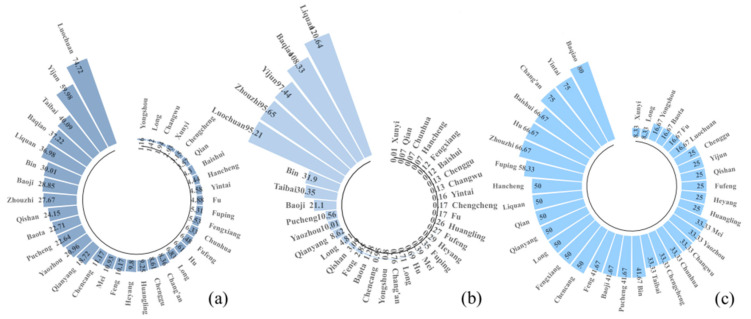
Changes in kiwifruit yield by county: (**a**) yield reduction rate (%), (**b**) yield reduction coefficient of variation and (**c**) the change in the probability occurrence of yield reduction rate (%).

Figure 8a–c show the yield reduction rate, the yield reduction coefficient of variation and the change in the probability occurrence of yield reduction rate, respectively. The counties with higher yield reduction rate were Luochuan (74.72%), Yijun (59.98%), Taibai (40.09%) and Baqiao (37.22%). The counties with higher yield reduction coefficients of variation were Liquan (120.64%), Baqiao (108.33%), Yijun (97.44%) and Zhouzhi (95.65%). The area with the highest probability occurrence of yield reduction rate was Baqiao, even reaching 85%. Baqiao, an emerging kiwifruit base county in Shaanxi Province, faces a higher risk of yield loss and is more affected by high-temperature disasters. Overall, kiwifruit yield was stable in all but a few counties. More than half of the areas have a probability occurrence of yield reduction rate of less than 50% and are less affected by meteorological disasters.

#### 4.2.2. Analysis of Spatial Patterns of High-Temperature Disaster Vulnerability

**Figure 9:**
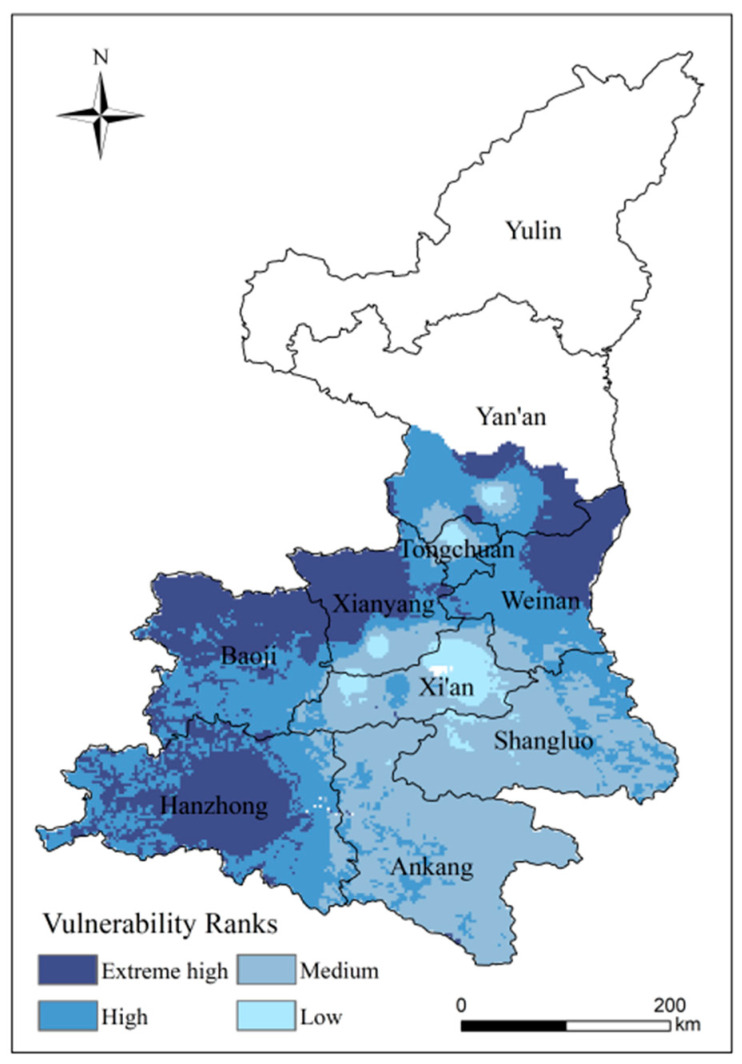
Distribution of vulnerability to kiwifruit in Shaanxi.

Areas of very extreme high, high, medium and low vulnerability represent 27.84%, 32.49%, 36.32% and 3.35% of the study area, respectively (Figure 9). The vulnerability of Hanzhong, the north of Baoji, the northwest of Xianyang and the east of Weinan is extremely high, and the ability to resist disaster in these areas is weak. Baoji, as the main producing area of kiwifruit in Shaanxi Province, has formed a centralized and continuous planting model of kiwifruit base county. If threatened by a high-temperature disaster, it can easily cause greater damage. Vulnerability in the southeast presents a gradually decreasing trend, and the eastern region in the study area is at low vulnerability. The Ankang and Shangluo kiwifruit planting areas have a lower degree of yield variability. Furthermore, these areas are at a higher altitude and close to the Qinling Mountains. The Qinling Mountains have a blocking effect on the warm and humid air currents from the south, making the nearby areas less affected by the high temperatures.

### 4.3. Comprehensive Assessment of High-Temperature Disaster Exposure

#### 4.3.1. Analysis of the Climatic Suitability of Kiwifruit

**Figure 10:**
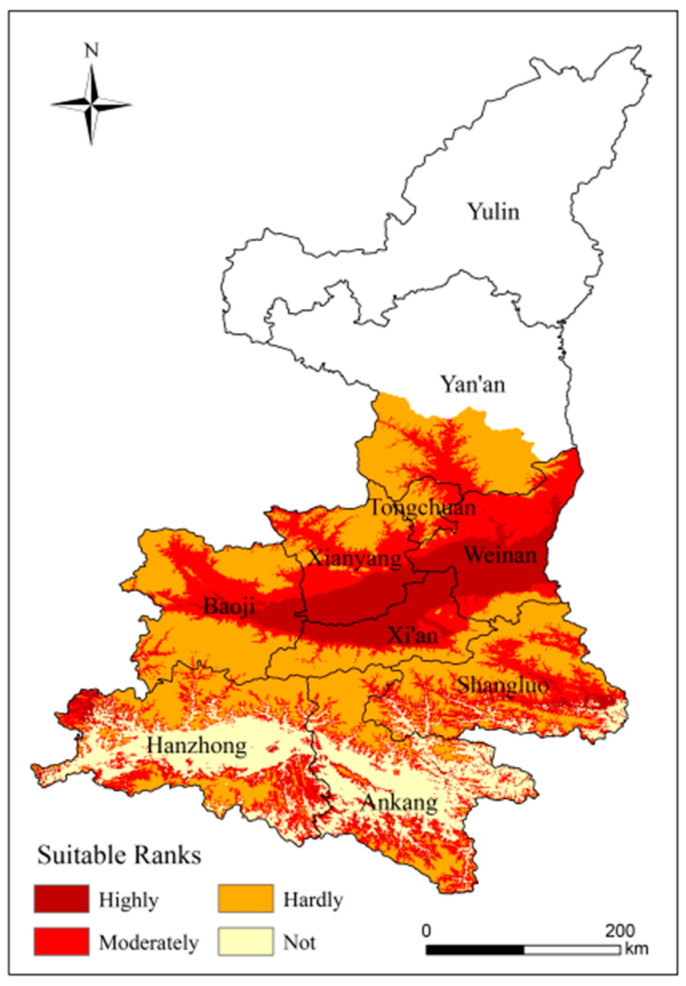
Climatic suitability zoning for kiwifruit in the study area.

Shaanxi kiwifruit planting areas are mainly distributed in the pre-mountain alluvium pro luvium fan area north of the Qinling Mountains, which has a warm temperate zone semi-humid and semi-dry climate. In recent years, with the quickening pace of rural industrial structure adjustment, the areas planted with kiwifruit and yield have continued to grow. A detailed climatic suitability zoning study is not only a reference for planting layout, but also a practical index of exposure (Figure 10). The highly suitable area is located in central Shaanxi, with a warm-temperate semi-humid and semi-dry climate, superior climatic resources, fertile soils and flat terrain. It accounts for 17.83% of the study area. The moderately suitable area extends from the highly suitable area outwards to below 1100 m above sea level in the Weibei Plateau and to higher elevations on both sides of the northern branch of the Qinling Mountains, and can be divided into two parts, north and south, accounting for 39.96% of the study area. In the north, temperatures are variable during spring, with more late frosts, less precipitation and more drought. There is abundant precipitation in the south, but they face higher temperatures. All areas are not very suitable for planting kiwifruit, except for Hanzhong and Ankang.

#### 4.3.2. Analysis of Spatial Patterns of High-Temperature Disaster Exposure

**Figure 11:**
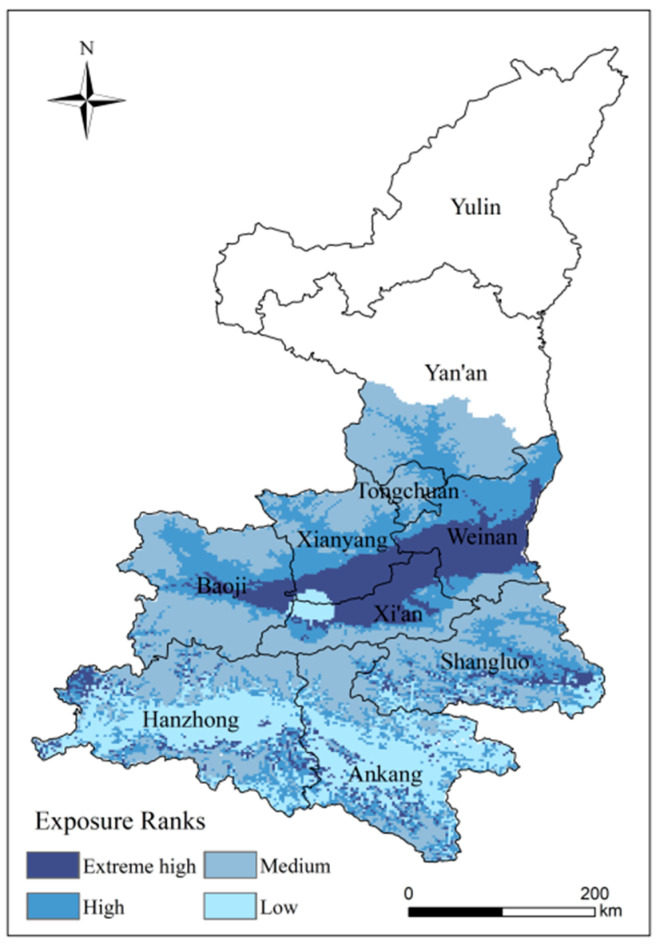
Distribution of exposure to kiwifruit in Shaanxi.

The exposed high-value area is located in the Guanzhong Plain region, including central Weinan, the part of Xianyang bordering Xi’an and Baoji, accounting for 14.54% of the study area (Figure 11). There is a large kiwifruit planting area in this area, and it has high climate suitability, so it is exposed. The central part of the study area has a climate suitable for the growth of kiwifruit and is suitable for extensive kiwifruit cultivation. It is also accompanied by a high potential risk. It is advisable to increase the cultivation of good kiwifruit varieties in the region and to strengthen pre-disaster prevention, response and post-disaster recovery in order to ensure the yield and quality of kiwifruit. Medium and low exposure areas represent 58.18% of the study area. Medium exposed areas are widely distributed and can affect kiwifruit yield if severe high-temperature events occur. Exposure in Hanzhong and Ankang is relatively low and potential losses from high-temperature disasters are likely to be low.

### 4.4. Comprehensive Assessment of High-Temperature Disaster Prevention and Mitigation Capacity

**Figure 12:**
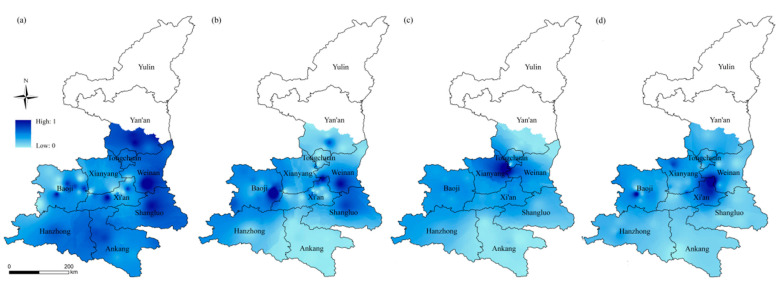
Spatial distribution of disaster prevention and mitigation capacity indexes: (**a**) total agricultural machinery power (KW), (**b**) fertilizer consumption (t), (**c**) per capita disposable income of farmers (yuan) and (**d**) rural electricity consumption per unit area (KW/h).

The total agricultural machinery power is high in the study area, with little difference between the north and south (Figure 12a). Weinan, Shangluo and Yan’an are at a high-value level, with a high level of agricultural modernization and therefore a high level of disaster resilience and relatively timely mitigation operations. In addition, the high total agricultural machinery power in the Shangluo region is related to the strong policy to develop agriculture in the region. The amount of fertilizer consumption can reflect the conditions of agricultural production in an area, and inputs of fertilizer to promote and improve crop growth can enhance crop resistance to disaster (Figure 12b). A comparison of the Figure 12a,b shows a certain consistency between fertilizer consumption and total agricultural machinery power. It shows a spatial distribution with more in the center and less in the north and south. Weinan, Shangluo, Yan’an and Baoji have relatively good agricultural production conditions. These areas are located in the plains and are suitable for cultivation and are relatively resilient to disaster. Per capita disposable income of farmers is another index of the comprehensive assessment of high-temperature disaster prevention and mitigation capacity (Figure 12c). Xianyang has a clear advantage in terms of disposing of funds and optimizing disaster prevention and mitigation measures. Rural electricity consumption per unit area gives an indication of the degree of modernization of the countryside and the affluence of farmers (Figure 12d). The high-value areas are Baoji, Yan’an and Xi’an. The low-value areas are Ankang and southeastern Hanzhong.

**Figure 13:**
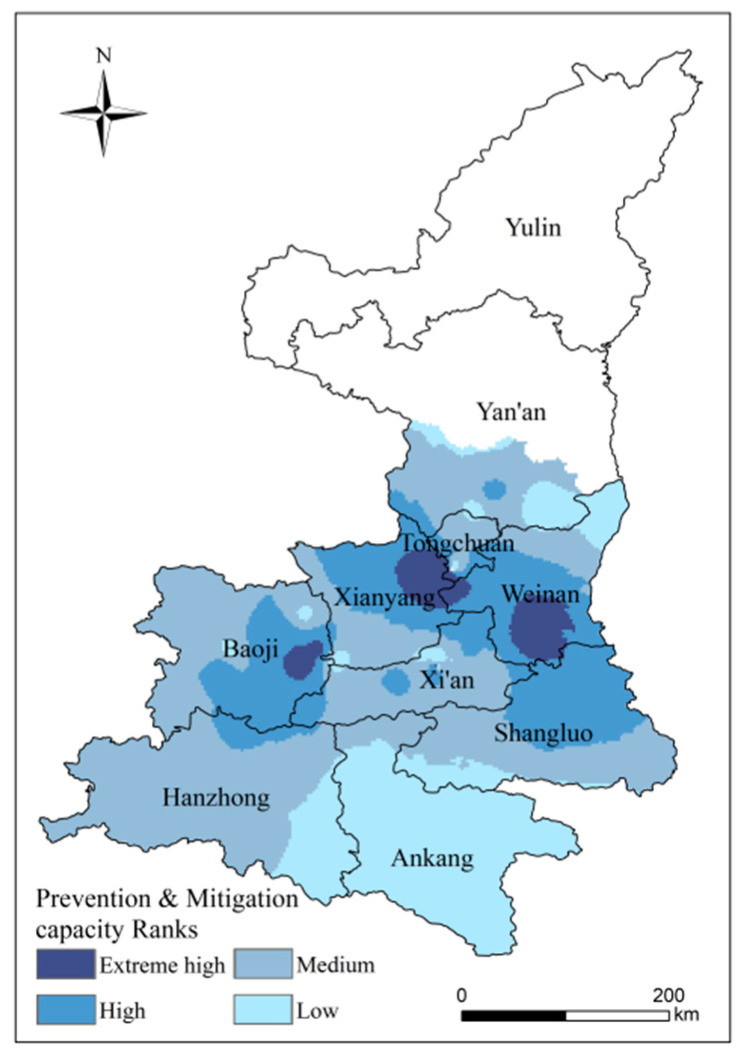
Distribution of high-temperature disaster prevention and mitigation capacity.

The study area has an overall medium level of disaster prevention and mitigation capacity, accounting for 47.43% (Figure 13). Ankang has a weak capacity for disaster prevention and mitigation, and its resistance and recovery from disaster need to be improved. The southern part of Weinan, the northeastern part of Xianyang and the central-eastern part of Baoji have extreme high disaster prevention and mitigation capacities, but this part of the area only accounts for 4.19% of the total area of the study area. The central part of the study area has a strong capacity for disaster prevention and mitigation, which is highly beneficial for fruit and food production, and have a better ability to cope with disaster.

### 4.5. Risk Assessment of High-Temperature Disasters

#### 4.5.1. Analysis of Spatial Patterns Risk of High-Temperature Disasters

**Figure 14:**
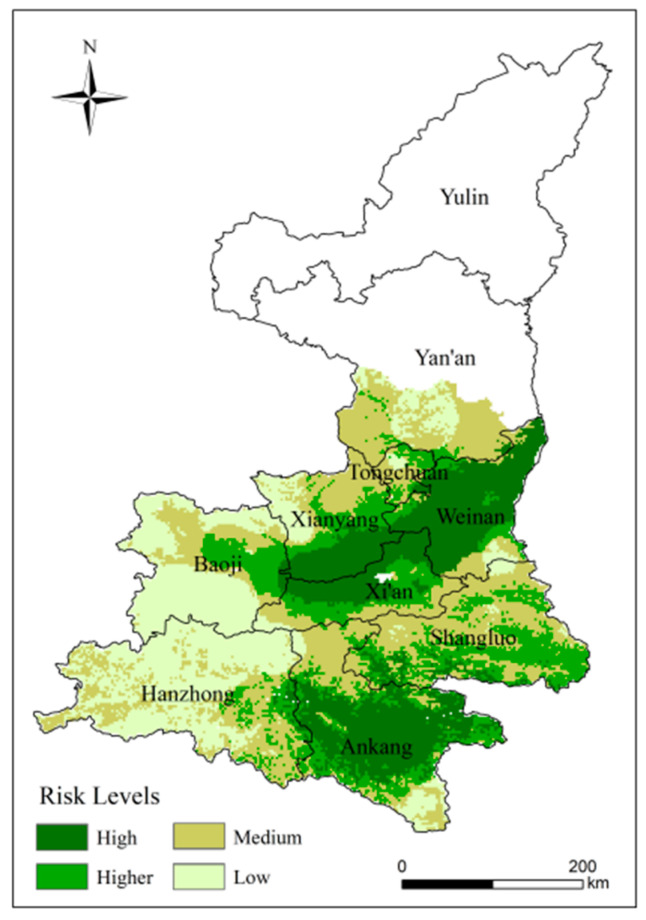
Distribution of high-temperature disaster to kiwifruit in Shaanxi.

We constructed risk assessment models using the results of hazard, vulnerability, exposure and disaster prevention and mitigation capacity. GIS was used to obtain results for high-temperature disaster risk assessment. The risk of high-temperature disasters is classified into four ranks from lowest to highest based on the optimal segmentation. Thus, the spatial distribution of high-temperature disaster risk was obtained (Figure 14).

The distribution of risk in kiwifruit planting areas shows clear regional differences. Extreme high-risk areas are concentrated in the central part of the study area and Ankang, accounting for 22.7% of the study area. These areas have a warm temperate monsoon climate with high temperature and rainy summer. The high-risk area spreads in a southerly and northerly direction, accounting for 19.22% of the study area. The medium risk area is located in the eastern part of the study area, accounting for 33.38%. The low level of risk areas covers Hanzhong and southern Baoji. This region is a major kiwifruit planting area and the low risk of high-temperature disasters is conducive to the development of kiwifruit farming.

#### 4.5.2. Validation of Risk Evaluation Results

To verify the reliability of the assessment results, the data of high-temperature events in Shaanxi Province from 1960 to 2020 were queried. Six of the more serious heat events were collected and collated (Table 6). The results of the study are more in line with historical disaster data, with the high-risk areas located in parts of central and southeastern Shaanxi. Weinan experienced extreme heat events in 1971 and 2014.

### 4.6. Research Limitations and Prospects

The climate in Shaanxi Province varies greatly, gradually from north to south to the temperate zone, warm temperate zone and north subtropical zone. With the comprehensive effect of complex geographical environment and changeable climatic environment, not just one type of meteorological disaster occurs in the region. Many kinds of disaster may exist at the same time and interact with each other. We selected only the most serious high-temperature disaster to kiwifruit for our risk assessment and did not consider other disasters (freezing injury in early spring, extreme precipitation, low temperature and sparse sunlight, etc.). Following studies can screen for different disaster intensities by counting the occurrences of disasters in each county over the years. We also recommend conducting comprehensive dynamic risk assessments of different regions, different growth periods and multiple disasters. In addition, unlike field crops, kiwifruit, as a highly centrally managed economic fruit, is not just passively influenced by the climate. Orchard management, cropping systems and cultivation practices will all have an impact, and can even actively adapt to environmental changes through human regulation. In future research, therefore, further consideration needs to be taken of park management and other factors that affect the final risk assessment.

### 4.7. Recommendations

As an important economic fruit in Shaanxi Province, kiwifruit faces the threat of extreme high temperature mainly during the summer, which directly affects its yield and economic efficiency. Based on the above research, we propose three recommendations. Firstly, as an economic fruit, kiwifruit planting patterns are heavily influenced by policy. It is recommended to increase government investment, adjust the industrial structure and select and breed varieties with higher resistance to reduce the impact of high-temperature disasters. Secondly, due to the economic benefits of kiwifruit, which have come to the fore in recent years, there has been a blind introduction of planting. It is recommended that planting is carried out according to the results of the climatic suitability zone and adjust measures to local conditions. Finally, we recommend strengthening orchard management and infrastructure, while enhancing professional training for fruit farmers and improving cultivation techniques. Furthermore, risk should be considered to minimize losses.

## 5. Conclusions

We are the first to present a study to assessment the risk of high-temperature disasters affecting the kiwifruit. A system and model for assessing the risk of high-temperature disaster to kiwifruit was constructed from four aspects: hazard, vulnerability, exposure and disaster prevention and mitigation capacity. At the same time, a study on the climatic suitability of kiwifruit for zoning was carried out and used as one of the indexes of exposure. The results are as follows:

(1) From five aspects, heat, light, water, soil resources and topography, 16 indexes were selected that have an important influence on the growth and development of kiwifruit, and a climate suitability zoning of kiwifruit in Shaanxi Province was carried out. We found that the areas of high suitability were located in the Guanzhong Plain region, including Weinan, southern Xianyang, northern Xi’an and central-eastern Baoji. The highly and moderately suitable area accounted for 42.21% of the study area.
(2) Areas at high risk of high-temperature disaster are located in Ankang, Weinan, southern Xianyang and northern Xi’an, accounting for 22.7% of the study area. As a relatively concentrated area of agricultural production in the plains, the risk of high-temperature disasters poses a significant threat to the region’s agricultural development. The south-western part of the study area has a low risk and is favorable to the kiwifruit industry.
(3) By comparing with historical disaster data, the areas where high-temperature disasters occurred are more consistent with our findings and the study has a high degree of confidence.

## Figures and Tables

**Table 1:** Classification of high-temperature disaster.

Period	Index	Disaster Level	Threshold
June–August	Daily maximum temperature (T_C_/°C)	Light	35 ≤ T_C_ < 38 (3–4 day)
		Moderate	35 ≤ T_C_ < 38 (5–8 day)
		Severe	35 ≤ T_C_ < 38 (≥9 day) or 38 ≤ T_C_ (≥2 day)

**Table 2:** Standard of climatic suitability regionalization index.

**Suitable Ranks**		Highly	Moderately	Hardly	Not
Heat Resource	Average annual temperature (°C)	[14, 16]	[13, 14)$\cup^{}$(16, 18]	[10, 13)$\cup^{}$(18, 20]	(−∞, 10)$\cup^{}$(20, +∞)
	Average temperature in January (°C)	[6, 8]	[4.5, 6)$\cup^{}$(8, 9]	[3.4, 5)$\cup^{}$(9, 10]	(−∞, 3)$\cup^{}$(10, +∞)
	Average temperature in March (°C)	[12, 14]	[11, 12)$\cup^{}$(14, 15]	[10, 11)$\cup^{}$(15, 16]	(−∞, 10)$\cup^{}$(16, +∞)
	Average temperature in July (°C)	[22, 24]	[20, 22)$\cup^{}$(24, 26]	[17, 20)$\cup^{}$(26, 28]	(−∞, 17)$\cup^{}$(28, +∞)
	≥10 °C accumulated temperature (°C)	[4500, 5200]	[4000, 4500)$\cup^{}$(5200, 5600]	[3500, 4000)$\cup^{}$(5600, 6000]	(−∞, 3500)$\cup^{}$(6000, +∞)
	Extreme minimum temperature (°C) (80% guaranteed)	[−3, +∞)	[–4, –3]	[–5, –4]	(−∞, −5)
	Average diurnal temperature amplitude in August (°C)	[8, 12]	[6, 8)	[5, 6)	(−∞, 5)
	Frost-free period (d)	[280, +∞)	[240, 280)	[200, 240)	(−∞, 200)
Light Resource	Annual sunshine hours (h)	[1200, 2000]	[900, 1200)$\cup^{}$(2000, 2200]	(−∞, 900)$\cup^{}$(2200, 2500]	(2500, +∞)
Water Resource	Annual average relative humidity (%)	[79, 82]	[75, 79)$\cup^{}$(82, 85]	[72, 75)	(−∞, 72)$\cup^{}$(85, +∞)
	Total annual precipitation (mm)	[1200, 1500]	[1100, 1200)$\cup^{}$(1500, 1600]	[900, 1100)$\cup^{}$(1600, 1700]	(−∞, 900)$\cup^{}$(1700, +∞)
Topography	Elevation (m)	[500, 1200]	[350, 500)$\cup^{}$(1200, 1500]	[200, 350)$\cup^{}$(1500, 2000]	(−∞, 200)$\cup^{}$(2000, +∞)
	Slope (°)	[10, 20]	[5, 10)$\cup^{}$(20, 25]	[3, 5)$\cup^{}$(25, 30]	(−∞, 3)$\cup^{}$(30, +∞)
	Slope direction	South/ Southeast/ Southwest	East/Northeast	West/Northwest	North
Soil Resource	Soil pH	[5.5, 6.5]	[5.0, 5.5)$\cup^{}$(6.5, 7.0]	[4.5, 5.0)$\cup^{}$(7.0, 7.5]	(−∞, 4.5)$\cup^{}$(7.5, +∞)
	Soil type	Sandy loamy	Light loamy/ medium loamy	Heavy loamy	Clay/loamy clay

**Table 3:** The range of ranks corresponding to the different classifications.

Suitable Ranks	Highly	Moderately	Hardly
Threshold	10–15	5–10	0–5

**Table 4:** Classification of suitability ranks.

Suitable Ranks	Highly	Moderately	Hardly	Not
Threshold	0.65–1	0.36–0.65	0.12–0.36	<0.12

**Table 5:** The weight of each risk assessment index.

Factor	Sub-Factor	Index	Weight
Hazard (H)	Disaster-inducing factors (0.832)	Light high-temperature disaster (XH1)	0.315
		Moderate high-temperature disaster (XH2)	0.324
		Severe high-temperature disaster (XH3)	0.361
	Formative Environment (0.168)	Gale days (XH4)	0.090
		Soil erosion (XH5)	0.078
Vulnerability (V)	Crop sensitiveness (S)	Yield reduction rate (XS1)	0.348
		Yield reduction coefficient of variation (XS2)	0.193
		Probability occurrence of yield reduction rate (XS3)	0.276
		Meteorological sensitiveness index (XS4)	0.183
	Adaptive capacity (A)	Soil organic matter (XA5)	0.561
		Soil total nitrogen content (XA6)	0.142
		Soil total phosphorus content (XA7)	0.139
		Soil total potassium content (XA8)	0.158
Exposure (E)	Climatic conditions	Climate suitability (XE1)	0.481
	Planting conditions	Planted area as a proportion of the province’s planted area (XE2)	0.519
Disaster prevention and mitigation capacity (C)	Agricultural production conditions factors	Total agricultural machinery power (XC1)	0.211
		Fertilizer consumption (XC2)	0.212
	Socioeconomic factors	Per capita disposable income of farmers (XC3)	0.361
		Rural electricity consumption per unit area (XC4)	0.216

**Table 6:** Historical disaster data for high temperature events in Shaanxi Province.

Sort	Time	Extreme Heat Event Records
1	1971.7.15–1971.7.28	From mid-July to mid-August 1971, there were high temperatures and little rain in Guanzhong and southern Shaanxi. The daily maximum temperature in the Weinan area was above 36 °C. The high temperature aggravated the drought and affected 133,000 hectares of farmland, accounting for 35 per cent of the total autumn field area. *
2	1972.8.7–1972.8.16	From late July to mid-August 1972, extreme heat events occurred in Hanzhong and southern Shaanxi. Cotton and maize were affected. *
3	1997.7.20–1997.7.27	From 1961 to 2006, the average number of high-temperature days in the northwest region was 2.4 days, with the most years being 1997, when there were 6.4 days. In Shaanxi Province, the annual number of high-temperature days is above 50, with daily maximum temperatures reaching 38–40 °C in Weinan and Xi’an. **
4	2001.7.11–2001.7.23	In mid to late July 2001, most areas in the middle and lower reaches of the Yangtze River and north of it in China experienced persistent extreme heat. The maximum temperature or the number of high-temperature days in many areas of southern Shaanxi Province exceeded the extreme values for the same period in history. The high temperatures and low rainfall were extremely detrimental to the growth of kiwifruit, citrus, grapes, apples and walnuts. ***
5	2014.7.27–2014.8.5	From 4 July to 10 August 2014, the longest consecutive run of high-temperature days in south-central Shaanxi was generally 10 days or more, reaching or exceeding historical extremes. Extreme high temperatures above 40 °C were experienced in local areas of Shaanxi. Intermittent hot weather occurred in southern Shaanxi, with Weinan reaching 39.1 °C. ****
6	2016.8.12–2016.8.21	July 20–August 26, a total of 30 provinces (autonomous regions and municipalities), 1653 counties (cities), had daily maximum temperatures of more than 35 °C high-temperature weather; the southeastern region of Shaanxi had amaximum temperature of 38–41 °C; Shaanxi Xunyang had one of up to 43.6 °C; 64 counties (cities) surpassed the local historical extreme; 11 provinces (autonomous regions and municipalities) in the south had an average number of high-temperature days of 19 days, the highest value since 1961. ****

* China Meteorological Disaster Dictionary—Shaanxi Volume. Wen Kegang. China Meteorological Press. ** Atlas of disastrous weather and climate in China (1961–2006). China meteorological administration. China Meteorological Press. *** Regional extreme events of drought, heavy precipitation, high and low temperatures in China. Ren Fumin. China Meteorological Press. **** China Meteorological Disaster Yearbook. China meteorological administration. China Meteorological Press.

## Data Availability

The data presented in this study are available upon request from the corresponding author.
